# Quantifying resource sharing in working memory

**DOI:** 10.3758/s13423-024-02494-4

**Published:** 2024-03-22

**Authors:** Julie Pougeon, Valérie Camos, Clément Belletier, Pierre Barrouillet

**Affiliations:** 1 Faculté de Psychologie et des Sciences de l’Éducation, Université de Genève, 40 Boulevard du Pont d’Arve, 1200 Genève 4, Switzerland; 2 Département de Psychologie, Université de Fribourg, Rue P.-A. de Faucigny 2, 1700 Fribourg, Switzerland; 3 Université Clermont-Auvergne, Clermont-Ferrand, France

**Keywords:** Working memory, Dual-task performance

## Abstract

Several models of working memory (WM), the cognitive system devoted to the temporary maintenance of a small amount of information in view of its treatment, assume that these two functions of storage and processing share a common and limited resource. However, the predictions issued from these models concerning this resource-sharing remain usually qualitative, and at which precise extent these functions are affected by their concurrent implementation remains undecided. The aim of the present study was to quantify this resource sharing by expressing storage and processing performance during a complex span task in terms of the proportion of the highest level of performance each participant was able to reach (i.e., their span) in each component when performed in isolation. Two experiments demonstrated that, despite substantial dual-task decrements, participants managed to preserve half or more of their best performance in both components, testifying for a remarkable robustness of the human cognitive system. The implications of these results for the main WM models are discussed.

Baddeley (2007) has defined working memory (WM) as a capacity-limited system devoted to the temporary maintenance and processing of a small amount of information during ongoing cognition. This need to concurrently fulfill the two functions of processing and storage has naturally raised the question of the structural and functional characteristics of such a system. Accordingly, Baddeley and Hitch’s (1974) seminal investigations aimed at establishing whether or not short-term memory could be considered as a plausible candidate for the role of WM by assessing to which extent a memory load impacted performance on a concurrent processing task like reasoning. This first investigation and the following studies by Baddeley and his colleagues led to the well-known multicomponent model (MCM), in which different structures are in charge of processing on the one hand and storage on the other, structures fueled by distinct pools of resource (Baddeley, 1986; Baddeley & Logie, 1999; Logie, 2011, 2018). According to this view, in dual tasks, storage should have a very limited or no impact on concurrent processing, and, vice versa, processing should not disrupt concurrent maintenance, a prediction buttressed by empirical evidence (e.g., Duff & Logie, 2001). Contrary to the early view, the more recent version of the MCM (Baddeley et al., 2021), predicts interference between processing and storage due to the involvement of the central executive in both activities. Other models would also predict dual-task costs because they assume that processing and storage either take place within a common mental space (Case, 1985; Case et al., 1982), share a common and limited resource like attention as in Cowan’s embedded-process model (Cowan, 1999, 2005; Cowan et al., 2021), or rely on a common supply on a temporal basis as in the time-based resource-sharing model (TBRS; Barrouillet & Camos, 2015, 2021). All these models assuming some resource sharing between processing and storage when concurrently performed predict reciprocal dual-task costs that have been reported in several studies (e.g., Barrouillet et al., 2004, 2011; Belletier et al., 2021; Chen & Cowan, 2009; Vergauwe et al., 2014). However, despite these pieces of evidence of a reciprocal detrimental effect between processing and storage when concurrently performed, the amplitude of the dual-task costs revealing resource sharing remains underspecified, and consequently so it is for the extent the presumed common resource is shared.

Resource sharing is usually tested through the search of dual-task deficits when comparing single and dual-task conditions, or when varying the difficulty of one task and assessing the effects on the other task (Baddeley & Hitch, 1974; Barrouillet et al., 2004; Vergauwe et al., 2014). However, although these paradigms allow to measure variations in performance, they cannot tell us to what extent a given observed variation is important or not for a given individual. Indeed, the same tasks and levels of difficulty being usually presented to all the participants who necessarily differ in capacities and optimal level of performance, it is difficult to assess the magnitude of the observed effects for a given individual or for a group. This point is of importance because, if an absence of dual-task decrement clearly points toward distinct systems and pools of resource, reciprocal dual-task costs between processing and storage can reveal either that both functions entirely rely on a common resource, or that these functions are in fact largely independent from each other, drawing only partially on a common and shared resource. The aim of this study was to address this question by quantifying the dual-task costs occurring between processing and storage in WM tasks in terms of the proportion of their optimal performance level individuals are able to preserve when performing concurrently the two tasks.

For this purpose, we asked participants to perform a WM complex span task in which they maintained series of letters for further serial recall, each letter being followed by a parity task on digits appearing successively on screen (Fig. 1). After having assessed the optimal performance (i.e., the span) of each participant on both storage and processing through a titration procedure, the two components set at span were combined in the complex span task, performance on storage and processing being measured for each individual in terms of percentage of their span for both components. Thus, besides replicating reciprocal dual-task costs between processing and storage, we were able to quantify a potential resource sharing between the two functions. When adding storage and processing performance expressed in percentage of their respective span, a total approaching 200% would reveal a large independence between the two functions, whereas a total tending towards 100% would reveal an increasingly complete sharing of a common resource. Under the hypothesis of linear functions relating the amount of resource invested to the level of performance for both storage and processing, a perfect resource sharing should result in a total that does not exceed and is even lower than 100% if coordinating the two tasks involves some cognitive cost.

**Fig. 1:**
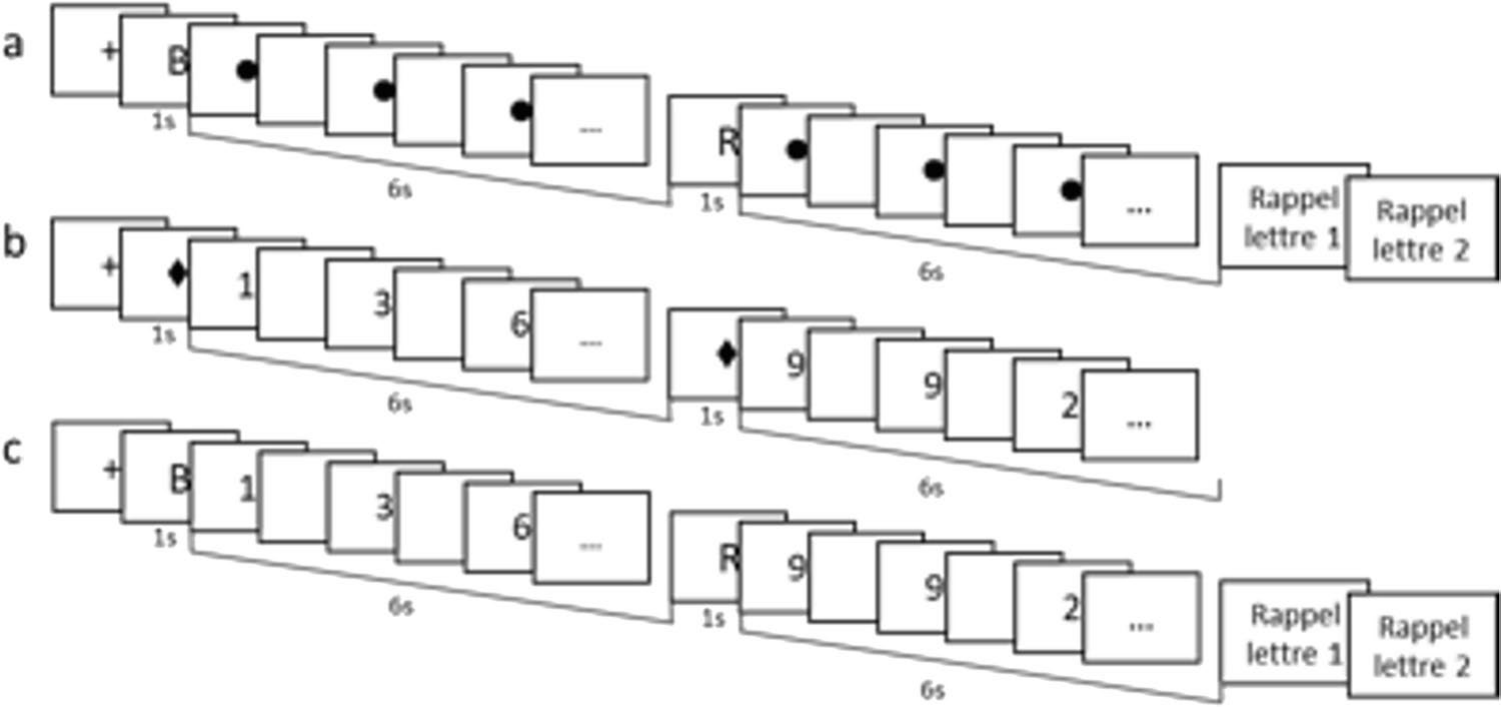
Illustration of the single memorization of letters task (**a**), single parity judgment task (**b**), and the dual task (**c**) in both experiments. The participants were instructed to fixate on the circle and diamond placeholders during the single tasks

## Experiment 1

### Method

#### Participants

Twenty-four undergraduate students (21 females; three males) between 18 and 21 years of age (*M* = 18.88 years, *SD* = 0.90) from the University Clermont Auvergne (France) received course credits for their participation. They all had normal or corrected-to-normal vision. We chose the sample size from previous experiments that provided conclusive evidence in support of dual-task costs (Belletier et al., 2021; Doherty & Logie, 2016). This study received approval from the ethics and research committee of the University Clermont Auvergne (IRB00011540-2022-82). Before beginning the tasks, participants were required to read a consent form, which informed them that the purpose of the experiment was “to study our ability to memorize and process information in a short period of time.”

#### Material

Tasks were administered using the PsychoPy 3.8 software (Peirce et al., 2019), and participants used the keyboard to complete the tasks. Yellow and green stickers were placed on the left and right directional keys, respectively, for the parity task. The memoranda were all the consonants except for “w” which is trisyllabic in French, “y” which is a vowel in some languages, and “z” due to its different position between QWERTZ and QWERTY keyboards. After performing the tasks, participants filled out an online questionnaire. The experimenter (the first author) remained in the experimental room during the entire experiment, sitting in such a way she could not see the computer screen.

#### General procedure

The experiment began with two titration procedures designed to measure participants’ memory and processing spans (i.e., the maximum number of letters they were able to memorize in an immediate serial recall task and the maximum number of digits the parity of which they were able to judge in a fixed period of time). Next, they performed single storage and processing tasks with a number of items to be memorized or processed equal to their spans. Finally, these two tasks were combined into a complex span task in which each memory item was followed by a phase of parity judgment. At the end of the experiment, participants completed a short questionnaire about the perceived difficulty of the task and the component of the complex span task they prioritized.

#### Titration on memory

Each letter appeared on screen for 1 second and was followed by a 6-second interval filled with a placeholder replacing the secondary task (a circle flickering at the centre of the screen; see Fig. 1a). After the last 6-second interval, participants recalled the letters by typing them on the keyboard in their order of presentation. For this purpose, the instruction “Recall Letter 1” was displayed on screen. The selected letter appeared for 500 ms and was replaced by “Recall Letter 2,” and so on until the end of the recall. Participants were asked to press the letter “O” for each forgotten letter.

The titration followed a staircase procedure with two trials per step. Beginning with four letters, one letter was added to the subsequent step if the participant succeeded to recall correctly 90% of the letters in a given step, or removed if this criterion was not reached. The titration procedure ended after eight steps of two trials. However, if the highest performance was achieved on the eighth step, the procedure continued until the participant failed. Memory span was the highest number of letters for which the 90% criterion was reached. This titration was preceded by three training trials with three letters.

#### Titration on processing

This titration on processing aimed at measuring the maximal number of digits the parity of which participants were able to judge in a series of 6-second intervals. The number of these intervals was equal to participant’s memory span. Following the same staircase procedure as for memory with two trials per step, titration started with four digits per 6-second intervals, this number being increased or decreased by one unit per step following the 90% correct criterion. A diamond placeholder replacing the letters was displayed for 1 s before each 6-second interval (Fig. 1b). The interstimuli interval after each digit being of 250 ms, each digit was displayed on screen for a number of ms equal to 6,000/*n* – 250, with *n* being the number of digits to be presented. Processing span corresponded to the highest number of digits per 6-second intervals for which the 90% criterion was reached. This titration was preceded by three training trials with three intervals of three digits to judge.

#### Single and dual tasks

After the two titrations, participants performed the memory and the processing tasks in isolation with five trials for each task. The number of items to be memorized and the number of digits presented in the 6-second intervals were equivalent to the memory and processing spans of each participant, as previously determined by the titration procedure. Subsequently, these storage and processing tasks were combined into a complex span task in which participants were required to memorize the letters while simultaneously judging the parity of the digits. Each letter was presented for 1 s, followed by a parity judgment interval of 6 s (Fig. 1c). After the last interval of parity judgment, participants had to recall the letters in their order of presentation. Each participant performed 10 trials of this complex span task.

#### Questionnaire

After completing the tasks, participants were asked to answer a questionnaire about their experience with the complex span task including questions about potential strategies of prioritization for storage and processing component (e.g., “In general, I put priority on the letters”) with a Likert scale from 1 (*never*) to 7 (*always*). The perceived difficulty of each component was assessed in the same way from “very easy” to “very difficult.” Additionally, there were two open-ended questions asking participants to describe their prioritization strategies and their understanding of the experiment goal.

#### Scoring

Participant’s accuracy in single and dual tasks was assessed through the percentage of letters recalled in correct serial position for the memorization task and the percentage of digits correctly judged in the parity judgment task. These percentages were corrected for guessing according to Diamond and Evans (1973) with *p*_*corr*_* = p*_*raw*_* – (p*_*errors*_*/(k-1))* where *p*_*corr*_ corresponds to the percentage corrected for guessing, *p*_*raw*_ to the percentage of correct responses, *p*_*errors*_ to the percentage of errors without counting omissions and *k* the number of possible responses, which was 18 for the memory task (the consonants of the alphabet excluding *w*, *y*, and *z*) and 2 for the parity task (even and odd).

#### Statistical analyses

Bayesian sample *t* tests and Bayesian repeated-measures analyses of variance (ANOVAs) were conducted with JASP (Version 0.18.3; JASP Team, 2024) using the defaults prior distributions parameters (Morey & Rouder, 2015; Rouder et al., 2009, 2012). The analyses were compared with the null model to get a BF_10_, which gives the strength of the data in favour of the hypotheses and determine the winning model in the Bayesian ANOVA. In the analyses of the model retained by the interactions, the BF_inclusion_ indicate the main and interaction effects. BF_10_ between 1 and 3 were interpreted as reflecting “anecdotal” evidence in favour of the alternative over the null hypothesis, which is not in favour of either model, between 3 and 10 as “moderate,” and between 10 and 30 as “strong” evidence in favour of the alternative hypothesis (Wagenmakers et al., 2018).

### Results

The mean memory span measured by the titration procedure was 6.42 letters (95% CI [5.90, 6.93]), and the mean processing span was 5.88 digits (95% CI [5.37, 6.38]) correctly judged per 6-second intervals.

Dual-task costs were assessed using Bayesian paired-sample *t* tests comparing single and dual tasks. As expected, memory accuracy was higher in the single (*M* = .88, 95% CI [.82, .93]) than dual task (*M* = .66, 95% IC [.58, .73]), BF_10_ = 4.80×10^2^, corresponding respectively to 5.62 and 4.17 letters recalled in correct serial position. Similarly, for processing accuracy, participants performed better in the single (*M* = .83, 95% CI [.80, .86]) than the dual task (*M* = .58, 95% CI [.53, .63]), BF_10_ = 2.06×10^7^, with respectively 4.87 and 3.39 digits correctly judged per 6-seconds intervals. Contrary to previous studies (Doherty et al., 2019), no evidence was gathered for a larger dual-task decrement in memory than processing as testified by the BF_inclusion_ of the interaction between tasks (single vs. dual) and component (memory vs processing), BF_inclusion_ = 0.46. In line with the slightly higher performance in the memory than the processing component of the complex span task (66% and 58%, respectively), participants declared stronger priority for storage than processing (sign test *z* = 3.27, *p* = .001), without any difference in perceived difficulty (sign test *z* = 0.63, *p* = .53).

The mean combined processing-storage performance in the complex span task came to 124% (66% for storage and 58% for processing, 95% CI [114, 134]). Bayesian one-sample *t* tests provided strong evidence that this combined performance was not inferior to 100%, BF_01_ = 20.18, and even superior, BF_10_ = 9.29×10^2^, but lower than 200%, BF_10_ = 2.08×10^11^.

### Discussion

The results of this first experiment showed that strong dual-task decrements affect both components of the complex span task, suggesting that processing and storage share some common resource. These results are consequently at odds with any model assuming that processing and storage are fuelled by distinct resources or supported by independent systems. Nonetheless, participants managed to preserve a substantial part of their optimal performance in both components, their combined performance being higher than 100%. This finding does not correspond to what could be expected from models assuming that both functions share a unique and common resource like the total processing space in Case’s (1985) model. However, several models suggest that verbal maintenance relies at least in part on a phonological or articulatory loop conceived as independent from the resource or system supporting processing (Baddeley, 1986; Baddeley et al., 2021), including models assuming a resource sharing between processing and storage like the TBRS (Barrouillet & Camos, 2021) or the embedded-process (Cowan et al., 2021) models. However, according to these latter models, blocking the articulatory loop would lead to a perfect resource sharing between processing and storage, both functions relying in this case on a unique and common attentional resource. A second experiment tested this hypothesis.

## Experiment 2

### Method

#### Participants

Forty-eight undergraduate students (44 females; four males) between 18 and 29 years of age (*M* = 19.33 years, *SD* = 1.92) from the University Clermont Auvergne (France) received course credits for their participation. None of them took part in Experiement 1, but were recruited in the same way and read the same forms before participating.

#### Materials and procedure

The materials and procedure were the same as in Experiment 1, except that all the tasks were performed under concurrent articulation. Participants were instructed to start uttering the syllables “*ba bi bou*” when the ready signal (a cross) appeared on screen before the first letter or diamond placeholder, and to keep uttering these syllables until the appearance of the prompt “Recall Letter 1” (Fig. 1). To prepare participants to perform this articulation at a regular pace, one beep sounded every second before each new task, indicating that participants should say one syllable per second.

### Results

The mean spans were 3.77 letters (95% CI [3.42, 4.12]), and 5.73 digits correctly judged per 6-second intervals (95% CI [5.37, 6.09]) for storage and processing, respectively.

As in Experiment 1, memory and processing accuracy were higher in the single than the dual task condition, revealing strong dual-task decrements. For memory, respectively, *M* = .88, 95% CI [.85, .91], and *M* = .45, 95% /ci [.41, .50]), BF_10_ = 1.59×10^18^, corresponding to 3.33 and 1.73 letters correctly recalled. For processing, *M* = .84, 95% CI [.82, .87], and *M* = .61, 95% CI [.58, .65]), BF_10_ = 6.78×10^14^, corresponding to 4.84 and 3.48 digits correctly judged per 6-second interval. However, contrary to the previous experiment, the Bayesian repeated-measures ANOVA revealed an interaction between tasks (single and dual) and components (storage and processing), BF_inclusion_ = 2.24×10^8^. Although there was no clear evidence for a difference between storage and processing in single tasks, BF_10_ = 2.04, participants performed better in processing than in storage in the dual task, BF_10_ = 9.27×10^3^. Questionnaire did not reveal that one task was significantly prioritized over the other (sign test *z* = 1.6, *p* = .09) though processing was perceived more difficult (sign test *z* = 3.92, *p* < .001).

**Fig. 2:**
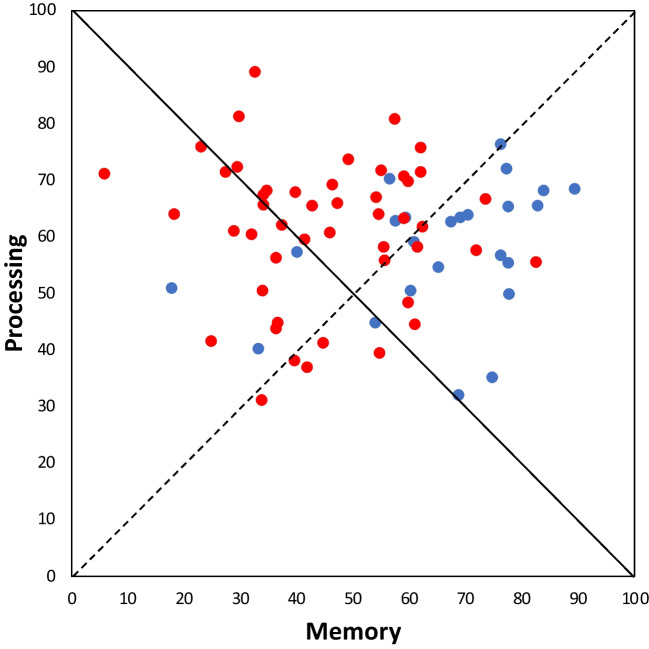
Distribution of participants as a function of their processing and storage scores in percentage of their span performance for Experiment 1 (blue dots) and Experiment 2 (red dots). The black diagonal corresponds to a sum of 100. The dotted diagonal materializes what would be the equality between the two scores. (Color figure online)

To understand the decline in combined processing-storage performance that dropped from 124% in Experiment 1 to 107% in Experiment 2 (45% for memory and 61% for processing, 95% CI [101, 112]), we assessed the effect of concurrent articulation through a Bayesian ANOVA, with experiments (1 vs. 2) and components (storage vs. processing) as factors. The analysis favoured the full model (BF_10_ = 4.33×10^6^) over any other model without interaction, for which existed strong evidence (BF_inclusion_ = 1.84×10^4^). Bayesian independent-sample *t* tests indicated that memory score strongly decreased from Experiment 1 (66%) to Experiment 2 (45%, BF_10_ = 4.89×10^3^), whereas there was anecdotal evidence for a stability of the processing score (from 58% to 61%, BF_10_ = 0.41; Fig. 2). Finally, there was strong evidence that this combined performance was not inferior, BF_01_ = 19.50, and even slightly superior to 100%, BF_10_ = 3.19, and of course still lower than 200%, BF_10_ = 2.93×10^18^.

### Discussion

In line with the hypothesis of a domain-specific and independent system for verbal maintenance (i.e., the articulatory loop), introducing a concurrent articulation led to a strong reduction of the proportion of verbal performance participants were able to maintain in the dual task (from 66% of memory span in Experiment 1 to 45% in Experiment 2), while processing performance remained roughly unchanged (from 58% to 61%). This revealed a more pronounced resource sharing, suggesting that maintenance of verbal information outside the articulatory loop competes with processing.^1^ Consequently, the combined processing-storage performance moved toward 100% (from 124% to 107%). However, it did not fall under this threshold contrary to what the hypothesis of a perfect trade-off would have predicted. In the following, we confront the main models of WM with the present findings.

## General discussion

The processing-storage dual function of WM raises the question of how these two functions coordinate, and more precisely of the degree of their mutual dependence (or independence) in terms of systems and resources that these two functions could share. Quantifying the potential resource sharing that several WM models assume seemed to us a modest but essential step towards elucidating this question. To the best of our knowledge, the present study is the first attempt in this direction using a complex span task, which is since the seminal work of Daneman and Carpenter (1980) a privileged tool for studying WM functioning and capacity. The methodology we chose was to measure the proportion of their maximal storage and processing capacity individuals were able to preserve when concurrently performing the two tasks in a WM complex span task.

First of all, we observed strong dual-task decrements incompatible with any model assuming independence between the two functions like the earliest versions of Baddeley’s (1986; Baddeley & Logie, 1999), or Logie’s MCM (Doherty & Logie, 2016). These strong decrements indicate that the two functions share some limited resource or supply, or at least that they interfere with each other as assumed in the latest version of the MCM (Baddeley et al., 2021). However, the quantification of this resource sharing revealed that a large part of individuals’ capacities is preserved. Even when a concurrent articulation prevented the use of the articulatory loop, the combined processing-storage performance exceeded what a simple resource-sharing model assuming the perfect sharing of a unique resource would have predicted, like in Case’s (1985) model where processing and storage share a limited total processing space, or in the TBRS model (Barrouillet & Camos, 2021) in which they alternate for occupying an executive loop that constitutes a central bottleneck. In both cases, it could be expected that storage and processing performance would be commensurate with the portion of space or time allocated to each function, the combined performance never exceeding 100%. Note that this combined performance could even be so much lower than 100% if the coordination of the two tasks, or the alternation of one to the other and the resulting switching costs, consume additional resources or time. However, the combined processing-storage performance in Experiment 2 was not inferior to 100%. There are several ways in accounting for the fact that the combined processing-storage score in Experiment 2 did not reflect what the strict sharing of a unique and common resource would predict.

First, it could be imagined that both functions draw on different resources and supplies with only a part of them being recruited by both functions. Such an option, developed by Logie and colleagues in their multi-component model (Logie, 2011; Logie et al., 2021), assumes that overall capacity of WM arises from multiple domain-specific memory systems and cognitive functions acting in concert. Such a view would probably not predict an overall performance reflecting a strict and perfect resource sharing although it is compatible with large dual-task decrements. However, the fact that the systems and functions recruited by a given activity remain underspecified in the model makes quantitative predictions difficult. In the same way, other models assume a resource sharing that differs from the spatial or temporal share evoked above without having strong commitments about the magnitude of this resource sharing. This is the case of Cowan’s (2005; Cowan et al., 2021) model that specifies the limitations of the storage function to a four-slot focus of attention, but not the way processing would consume this attentional resource. This seems also to be the case of Engle’s approach of WM capacity as controlled attention (Engle et al., 1999; Mashburn et al., 2021; Shipstead et al., 2016). Processing and storage functions are certainly fuelled by this controlled attention, but the way this sharing occurs seems to be out of the scope of the model. Thus, models endorsing a multicomponent view of WM or considering attention as a kind of energy fuelling both processing and storage cannot be considered at odds with our findings, but make few precise quantitative predictions about how resource sharing would affect performance.

Second, it could be the case that the processing-storage resource sharing occurs on a spatial, as in Case’s (1985) model, or temporal basis, as in the TBRS model (Barrouillet & Camos, 2021), while resulting in a combined performance exceeding 100%. This would be possible if the relation between the amount of resource invested and performance is not linear, but follows some power function, performance rapidly increasing with the first units of resource invested, and then gradually levelling out. Concerning the resource-performance function, Case et al. (1982) have provided several examples of a linear relation between processing efficiency, which is assumed to determine the mental space occupied by this processing, and memory span. In the same way, it can be expected that the number of digits processed in the present task varies linearly with the time allocated to this activity. Moreover, several studies suggest that the relation between the amount of time available for maintenance activities and memory performance in WM complex span tasks is also linear (Barrouillet et al., 2007, 2011). Thus, empirical data are still lacking for buttressing the hypothesis of a nonlinear function between resource and WM performance. Another possibility would be that the relation between the invested resource and memory performance is structurally limited in such a way that, once this limit is reached, additional resource investment would have no effect. In this case, even a strict sharing of a common resource would result in an overall performance exceeding 100%. We recently obtained results fitting this latter option (Barrouillet et al., 2024). Thus, the models that specify the shared resource as a mental space or a time for occupying a central bottleneck need additional assumptions in order to account for our observations.

Commenting the first studies about WM he conducted with Hitch (Baddeley & Hitch, 1974), Baddeley (1986) noted, “We found the cognitive system to be much robust than anticipated” (p. 69). Although the present results show that the effects of storage on concurrent processing are more pronounced than Baddeley and Hitch’s first investigations suggested, the conclusion of a surprising robustness of human cognitive system still holds. The present study, which provides a first attempt to quantify the resource sharing in WM, showed that even in the most demanding conditions, the human cognitive system manages to preserve more than half of its efficiency.

## Data Availability

The preregistration (https://osf.io/8xjz3), dataset, materials, and program code used on PsychoPy for stimuli presentation of the current study (https://osf.io/kp6f7) are available on OSF.

## Footnotes

1 It might be argued that articulatory suppression (AS) itself should be taken into account in quantifying resource sharing because it constitutes an additional process consuming the same resource shared by storage and processing. However, the consumption of this resource by AS is at best marginal. Indeed, adding AS made processing span only drop from 5.88 in Exp. 1 to 5.73 digits in Exp. 2. A Bayesian *t* test revealed a BF_10_ of 0.28, providing moderate evidence for the null, while the effect on storage span was huge, from 6.42 to 3.77 letters, with a BF_10_ of 2.60×10^9^. Thus, in line with the TBRS model, it can be assumed that AS hinders some of the memory maintenance system without significantly affecting processing resources.

## References

[CR1] Baddeley, A. D. (1986). *Working memory*. Clarendon Press.

[CR2] Baddeley, A. D. (2007). *Working memory, thought, and action*. Oxford University Press.

[CR3] Baddeley, A. D., & Hitch, G. J. (1974). Working memory. In G. A. Bower (Ed.), *Recent advances in learning and motivation* (8th ed., pp. 647–667). Academic Press.

[CR4] Baddeley, A. D., & Logie, R. H. (1999). Working memory: The multiple-component model. In A. Miyake & P. Shah (Eds.), *Models of working memory: Mechanisms of active maintenance and executive control* (pp. 28–61). Cambridge University Press.

[CR5] Baddeley, A. D., Hitch, G. J., & Allen, R. (2021). A multicomponent model of working memory. In R. H. Logie, V. Camos, & N. Cowan (Eds.), *Working memory: State of the science* (pp. 10–43). Oxford University Press.

[CR6] Barrouillet, P., & Camos, V. (2015). *Working memory: Loss and reconstruction*. Psychology Press.

[CR7] Barrouillet, P., & Camos, V. (2021). The time-based resource-sharing model of working memory. In R. H. Logie, V. Camos, & N. Cowan (Eds.), *Working memory: State of the science* (pp. 85–115). Oxford University Press.

[CR8] Barrouillet, P., Bernardin, S., & Camos, V. (2004). Time constraints and resource-sharing in adults’ working memory spans. *Journal of Experimental Psychology: General,**133*, 83–100. 14979753 10.1037/0096-3445.133.1.83

[CR9] Barrouillet, P., Bernardin, S., Portrat, S., Vergauwe, E., & Camos, V. (2007). Time and cognitive load in working memory. *Journal of Experimental Psychology: Learning, Memory and Cognition,**33*, 570–585. 17470006 10.1037/0278-7393.33.3.570

[CR10] Barrouillet, P., Portrat, S., & Camos, V. (2011). On the law relating processing and storage in working memory. *Psychological Review,**118*, 175–192. 21480738 10.1037/a0022324

[CR11] Barrouillet, P., Camos, V., Pougeon, J., Beaudet, J., Croizet, P., & Belletier, C. (2024). Human cognitive system privileges processing over short-term storage: Asymmetry in working memory limitations. *Journal of Experimental Psychology: Learning, Memory, and Cognition,**33*(3), 570–585. 10.1037/0278-7393.33.3.570 10.1037/xlm0001371 39052392

[CR12] Belletier, C., Camos, V., & Barrouillet, P. (2021). Is the cognitive system more robust than anticipated? Dual-task costs and residuals in working memory. *Journal of Experimental Psychology: Learning, Memory, and Cognition,**47*, 498–507. 33074693 10.1037/xlm0000961

[CR13] Case, R. (1985). *Intellectual development: Birth to adulthood*. Academic Press.

[CR14] Case, R., Kurland, M., & Goldberg, J. (1982). Operational efficiency and the growth of short-term memory. *Journal of Experimental Child Psychology,**33*, 386–404.

[CR15] Chen, Z., & Cowan, N. (2009). How verbal memory loads consume attention. *Memory & Cognition,**37*(6), 829–836. 19679862 10.3758/MC.37.6.829 PMC2804027

[CR16] Cowan, N. (1999). An embedded-process model of working memory. In A. Miyake & P. Shah (Eds.), *Models of working memory: Mechanisms of active maintenance and executive control* (pp. 62–101). Cambridge University Press.

[CR17] Cowan, N. (2005). *Working memory capacity*. Psychology Press.

[CR18] Cowan, N., Morey, C. C., & Naveh-Benjamin, M. (2021). An embedded-processes approach to working memory. In R. H. Logie, V. Camos, & N. Cowan (Eds.), *Working memory: State of the science* (pp. 44–84). Oxford University Press.

[CR19] Daneman, M., & Carpenter, P. A. (1980). Individual differences in working memory and reading. *Journal of Verbal Learning and Verbal Behavior,**19*, 450–466.

[CR20] Diamond, J., & Evans, W. (1973). The correction for guessing. *Review of Educational Research,**43*(2), 181–191. 10.3102/00346543043002181

[CR21] Doherty, J. M., & Logie, R. H. (2016). Resource-sharing in multiple-component working memory. *Memory & Cognition,**44*, 1157–1167. 27287373 10.3758/s13421-016-0626-7 PMC5085983

[CR22] Doherty, J. M., Belletier, C., Rhodes, S., Jaroslawska, A. J., Barrouillet, P., Camos, V., Cowan, N., Naveh-Benjamin, M., & Logie, R. (2019). Dual-task costs in working memory: An adversarial collaboration. *Journal of Experimental Psychology: Learning, Memory, & Cognition,**45*, 1529–1551. 30407025 10.1037/xlm0000668 PMC6727883

[CR23] Duff, S. C., & Logie, R. H. (2001). Processing and storage in working memory span. *Quarterly Journal of Experimental Psychology,**54*, 31–48. 10.1080/02724980042000011 11216320

[CR24] Engle, R. W., Kane, M. J., & Tuholski, S. W. (1999). Individual differences in working memory capacity and what they tell us about controlled attention, general fluid intelligence and functions of the prefrontal cortex. In A. Miyake & P. Shah (Eds.), *Models of working memory: Mechanisms of active maintenance and executive control* (pp. 102–134). Cambridge University Press.

[CR25] JASP Team. (2024). *JASP* (Version 0.18.3) [Computer software]. https://jasp-stats.org/. Accessed 21 Mar 2024.

[CR26] Logie, R. H. (2011). The functional organization and capacity limits of working memory. *Current Directions in Psychological Science,**20*, 240–245.

[CR27] Logie, R. H. (2018). Human cognition: Common principles and individual variation. *Journal of Applied Research in Memory and Cognition,**7*, 471–486.

[CR28] Logie, R. H., Belletier, C., & Doherty, J. M. (2021). Integrating theories of working memory. In R. H. Logie, V. Camos, & N. Cowan (Eds.), *Working memory: State of the science* (pp. 389–430). Oxford University Press.

[CR29] Mashburn, C., Tsukahara, J., & Engle, R. W. (2021). Individual differences in attention control: Implications for the relationship between working memory capacity and fluid intelligence. In R. H. Logie, V. Camos, & N. Cowan (Eds.), *Working memory: State of the science* (pp. 175–211). Oxford University Press.

[CR30] Morey, R. D., & Rouder, J. N. (2015). *BayesFactor* (Version 0.9.11-3) [Computer software].

[CR31] Peirce, J., Gray, J. R., Simpson, S., MacAskill, M., Höchenberger, R., Sogo, H., Kastman, E., & Lindeløv, J. K. (2019). PsychoPy2: Experiments in behavior made easy. *Behavior Research Methods,**51*(1), 195–203. 10.3758/s13428-018-01193-y 30734206 10.3758/s13428-018-01193-y PMC6420413

[CR32] Rhodes, S., Jaroslawska, A. J., Doherty, J. M., Belletier, C., Naveh-Benjamin, M., Cowan, N., Camos, V., Barrouillet, P., & Logie, R. H. (2019). Storage and processing in working memory: Assessing dual-task performance and task prioritization across the adult lifespan. *Journal of Experimental Psychology: General,**148*, 1204–1227. 30667263 10.1037/xge0000539 PMC6586477

[CR33] Rouder, J. N., Speckman, P. L., Sun, D., Morey, R. D., & Iverson, G. (2009). Bayesian *t* tests for accepting and rejecting the null hypothesis. *Psychonomic Bulletin & Review,**16*(2), 225–237. 10.3758/PBR.16.2.225 19293088 10.3758/PBR.16.2.225

[CR34] Rouder, J. N., Morey, R. D., Speckman, P. L., & Province, J. M. (2012). Default Bayes factors for ANOVA designs. *Journal of Mathematical Psychology,**56*(5), 356–374. 10.1016/j.jmp.2012.08.001

[CR35] Shipstead, Z., Harrison, T. L., & Engle, R. W. (2016). Working memory capacity and fluid intelligence: Maintenance and disengagement. *Perspectives on Psychological Science,**11*, 771–799. 27899724 10.1177/1745691616650647

[CR36] Vergauwe, E., Camos, V., & Barrouillet, P. (2014). The impact of storage on processing: Implications for structure and functioning of working memory. *Journal of Experimental Psychology: Learning, Memory,**40*, 1072–1095. 10.1037/a0035779 24564542

[CR37] Wagenmakers, E.-J., Marsman, M., Jamil, T., Ly, A., Verhagen, J., Love, J., Selker, R., Gronau, Q. F., Šmíra, M., Epskamp, S., Matzke, D., Rouder, J. N., & Morey, R. D. (2018). Bayesian inference for psychology. Part I: Theoretical advantages and practical ramifications. *Psychonomic Bulletin & Review,**25*(1), 35–57. 10.3758/s13423-017-1343-3 28779455 10.3758/s13423-017-1343-3 PMC5862936

